# Impinge Weyl advantages on light

**DOI:** 10.1038/s41377-023-01100-x

**Published:** 2023-03-02

**Authors:** Xiaomu Wang, Dong Sun

**Affiliations:** 1 School of Electronic Science and Engineering, Nanjing University, Nanjing, 210093 China; 2 International Center for Quantum Materials, School of Physics, Peking University, Beijing, 100871 China

**Keywords:** Optical physics, Electronics, photonics and device physics

## Abstract

Weyl semimetals are emerging topological materials with intriguing physical properties. Now this exotic matter may lead to novel photonic and optoelectronic applications.

Weyl semimetal refers to semimetal whose quasiparticle excitation is Weyl fermion^1^. In the context of solid-state physics, the elementary Dirac equation for massless fermion describes four bands touch at a point. Interestingly, a massless fermion can also obtain a definite ‘handedness’ by breaking the Dirac equation to two-component halves, resulting in two band-degenerate Weyl nodes with either left-handed or right-handed chirality (see Fig. 1a)^2^. The unique band dispersion makes Weyl semimetal as an emerging platform hosting tremendous exotic physical properties^3^. And the Weyl semimetals have been theoretically known for a long time and seen a great growth of experimental interests in recent years. Especially, the discovery of new Weyl materials and novel Weyl physics arouse from two two-band degenerate Weyl points have been clearly demonstrated in materials that break either inversion or time-reversal symmetry. Notably, an equally important step forward is to develop schemes for achieving quantum control of the Weyl quasiparticle excitations by optical methods. Vice versa, Weyl semimetals also provide new opportunities to impinge interesting Weyl physics on novel manipulation of light through unique interaction between light and Weyl Fermions. Now writing in eLight, Shanhui Fan group reviewed the basic concepts and optical responses of Weyl semimetals, and survey their applications in optics and thermal photonics^4^.

**Fig. 1:**
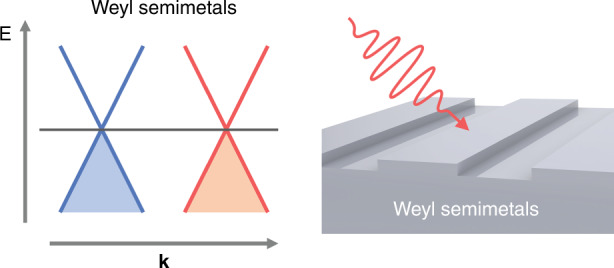
Typical band structure of Weyl semimetals (left) and its nano-structured photonic (right)

The chiral Weyl points have a topological meaning: each Weyl point can be assigned a topological number, directly related to the Chern numbers. This chirality leads to a marvelous phenomenon called chiral anomaly. Namely, when Weyl fermions purely with a single chiral coupled to electromagnetism, the conservation of charge can be broken^5^. There are always multiple Weyl points (so that the total charge cancels) but they may occur at different energies, resulting the semimetal is more conductive with an increasing magnetic field. This violation of the conservation of chiral charge leads to an axial charge current. In the Review, the authors discuss details of electromagnetic response in the presence of chiral anomaly. Key physics such as optical nonreciprocity are clearly analyzed. The substantial studies in fundamental physics paved a way toward novel applications. For example, chiral plasmons, unidirectional waveguide and non-magneto Faraday effect like those observed in twist bilayer graphene^6^ are proposed.

In a Weyl semimetal, Weyl nodes of opposite chirality are separated in momentum space and are connected only through the crystal boundary by an exotic non-closed surface state, known as the Fermi arcs. Experimentally, this Fermi arc surface states play as a crucial signature in the discovery of Weyl semimetal. It was the detection of these Fermi arcs in photoemission that gave solid evidence that the Weyl semimetal had indeed been realized^7,8^. The existence of Fermi arcs as surface states can also significantly alter the photo-response by modifying the boundary conditions of Maxwell equations. Intriguing photonic states such as Fermi arc plasmons are predicted in this Review. Remarkably, these optical responses would in turns enable improved observation of the Fermi arc surface states by means other than photoemission.

Emerging Weyl semimetals are also of particular scientific and technical importance for nonlinear optics, especially for response that arises directly from quantum geometry and topology of Weyl cones^9^. Corresponding to the topological charge (Chern number) of a chiral Weyl cone, the circular photogalvanic response of the cone is theoretically predicted to be quantized with the Chern number, which is a smoking gun optical signature to justify Weyl Fermions, but remains to be elusive experimentally. However, the experimental progresses are generally more advanced for nonlinear comparing to linear optical response, and some nonlinear optical effects have already reserved their unique applications in functional devices. An established example is the shift current response can be enhanced by the Berry phase at the vicinity of Weyl nodes^10^, such effect is directly applicable for high-performance photodetection and energy harvesting^11^. In addition, photodetectors based on Weyl semimetals exhibits orbital photogalvanic effect, applicable for direct detection of the orbital angular momentum of light, a very changeling task otherwise^12,13^. As exhausted in the review, other nonlinear effects, such as anomalous current, second and high harmonic generation, inverse Faraday effect, are actively explored experimentally and one can envision their applications in nonlinear optoelectronics.

Overall, the new topological phenomena of Weyl semimetals beyond topological insulators have made new physics accessible and signaled potential applications, despite the early stage of the research. Several exciting transport and optical phenomena that are still being sought in further experiments.
